# Integrating disparate datasets to model the functional response of a marine predator: A case study of harbour porpoises in the southern North Sea

**DOI:** 10.1002/ece3.8380

**Published:** 2021-11-30

**Authors:** Janneke M. Ransijn, Philip S. Hammond, Mardik F. Leopold, Signe Sveegaard, Sophie C. Smout

**Affiliations:** ^1^ Sea Mammal Research Unit Scottish Oceans Institute University of St Andrews St Andrews UK; ^2^ Wageningen Marine Research Wageningen University & Research Den Helder The Netherlands; ^3^ Department of Bioscience Aarhus University Roskilde Denmark

**Keywords:** multi‐species functional response, North Sea, *Phocoena phocoena*, predator–prey interactions, prey switching, sandeels

## Abstract

1. Quantifying consumption and prey choice for marine predator species is key to understanding their interaction with prey species, fisheries, and the ecosystem as a whole. However, parameterizing a functional response for large predators can be challenging because of the difficulty in obtaining the required data on predator diet and on the availability of multiple prey species.
2. This study modeled a multi‐species functional response (MSFR) to describe the relationship between consumption by harbour porpoises (*Phocoena phocoena*) and the availability of multiple prey species in the southern North Sea. Bayesian methodology was employed to estimate MSFR parameters and to incorporate uncertainties in diet and prey availability estimates. Prey consumption was estimated from stomach content data from stranded harbour porpoises. Prey availability to harbour porpoises was estimated based on the spatial overlap between prey distributions, estimated from fish survey data, and porpoise foraging range in the days prior to stranding predicted from telemetry data.
3. Results indicated a preference for sandeels in the study area. Prey switching behavior (change in preference dependent on prey abundance) was confirmed by the favored type III functional response model. Variation in the size of the foraging range (estimated area where harbour porpoises could have foraged prior to stranding) did not alter the overall pattern of the results or conclusions.
4. Integrating datasets on prey consumption from strandings, predator foraging distribution using telemetry, and prey availability from fish surveys into the modeling approach provides a methodological framework that may be appropriate for fitting MSFRs for other predators.

## Graphical abstract

A multi‐species functional response (MSFR) was modeled to describe the relationship between consumption by harbour porpoises and the availability of multiple prey species in the southern North Sea. Bayesian methodology was employed to estimate MSFR parameters and to incorporate uncertainties in diet and prey availability estimates. Results indicated a strong preference for sandeel in the study area and prey switching behavior was confirmed by the favored type III functional response model.

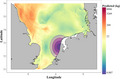

## 1 INTRODUCTION

Prey populations are directly and indirectly affected by predation and their dynamics are influenced by long‐term and short‐term responses of predators (Holling, 1959; Murdoch & Oaten, 1975). The functional response helps to assess the potential impact that predators could have on their prey by describing the response of predator consumption rates to varying prey densities, providing insight into prey preference and general predator–prey interactions (Dale et al., 1994). High consumption rates indicate strong interactions between predators and prey, resulting from high encounter rates and/or active predator choice. Switching between prey species may occur if predator preference changes with prey density, for example, when predators avoid scarce prey (Holling, 1959).

Although the functional response has been subject to extensive empirical research, most studies have been conducted within a laboratory setting or have described relationships among a small number of species (Morozov & Petrovskii, 2013). Modeling the multi‐species functional responses (MSFR) for wild animals is challenging because observing both consumption and prey availability outside a controlled environment is difficult. Parametrizing a MSFR requires substantial datasets on predator diet and distribution, and on the availability of multiple prey species covering a range of prey densities. It is not surprising, therefore, that the ecological role of most large predators has not been quantified and that we have an incomplete picture of their impacts in many ecosystems (Estes et al., 2011). However, the use of Bayesian methods can overcome the problem of data sparsity, allowing MSFR models to be fitted for top predators (Smout et al., 2014; Suryawanshi et al., 2017).

The aim of this study is twofold. Firstly, we develop a framework to integrate long‐term datasets on predator consumption, predator distribution, and prey abundance to model the MSFR of a marine high trophic level predator. The framework consists of a number of methodological steps for modeling changes in diet in relation to prey abundance, which are appropriate for mobile marine predators for which diet is sampled at specific locations. Our intention is that this methodological framework can serve as a model for other similar studies and thus help improve understanding of the ecological role of high trophic level marine predators. We develop and apply this framework using the harbour porpoise (*Phocoena phocoena*) in the southern North Sea as a case study to examine the methodology, model performance, model output, and the sensitivity of the results to variation in assumptions. We choose the harbour porpoise partly because there are data on prey consumption from the stomach contents of stranded porpoises in the Netherlands (Leopold, 2015), data on the distribution and movements of individual porpoises in the North Sea from satellite‐linked telemetry (Sveegaard et al., 2011), and data on prey abundance from the ICES International Bottom Trawl Surveys (ICES, 2018).

Secondly, in choosing the harbour porpoise as a case study, we aim to improve ecological understanding of an important marine predator in European Atlantic waters. The harbour porpoise is the most abundant large marine predator in the North Sea (Hammond et al., 2013) and its diet includes species that are also targeted by commercial fisheries (Santos & Pierce, 2003), such as whiting (*Merlangius merlangus*), Atlantic herring (*Clupea harengus*), and sandeels (Ammodytidae). Harbour porpoises have a high metabolic rate and only a limited energy storage capacity, which limits their ability to buffer against diminished food availability/quality and makes them more susceptible to starvation if they fail to meet their high metabolic demands (Rojano‐Doñate et al., 2018; Spitz et al., 2012). They have high ingestion rates and probably must consume prey on a daily basis (Kastelein et al., 2019; Wisniewska et al., 2016), unlike other cetaceans or pinnipeds that might move through certain areas while not foraging, and thus are particularly appropriate for this study. North Sea wide surveys showed a major north to south shift in the summer distribution of harbour porpoise from 1994 to 2005, maintained through 2016, which was likely linked to changes in prey distribution (Hammond et al., 2002, 2013, 2021). Information on the dynamic relationship between harbour porpoises and their prey is largely lacking but improving understanding of harbour porpoise predator–prey relationships may help to explain the observed shift in distribution.

## 2 MATERIAL AND METHODS

As a framework for analysis, the following sequence of steps (described in detail below) was followed to parameterize the functional response: (1) estimation of diet composition; (2) estimation of foraging range; (3) estimation of prey availability; and (4) fitting the multi‐species functional response. All data processing and modeling were performed in software R (R Development Core Team, 2018), and MSFR fitting was carried out in WinBUGS (Lunn et al., 2000).

### 2.1 Data preparation

#### 2.1.1 Diet composition

To obtain information on harbour porpoise prey consumption, diet composition was estimated from the hard remains of prey (fish otoliths) recovered from the stomachs of individual animals stranded along the Dutch coastline between 2006 and 2015. To match diet composition to availability of prey data strandings that occurred in November–April were assigned to quarter 1 and those in May–October to quarter 3 (see Section 2.1.3). Sample collection and analysis are described in Leopold (2015). Postmortem examinations were carried out on stranded animals documenting standard measurements (e.g., body length). Prey species were identified to the lowest possible taxon. Otoliths were measured, paired when possible, and graded for wear. Grade‐specific correction factors were used to estimate undigested otolith size and prey weight was estimated by applying otolith size–fish mass relationships. Prey species that contributed ≥5% of the total estimated prey weight were selected as main prey species.

Uncertainty in diet composition arises from measurement (estimation of prey weight) and sampling error (Hammond & Rothery, 1996). Sampling error was estimated by nonparametric bootstrapping using individual stranded porpoises as the sampling unit, stratified by season. To balance carcass freshness and retain an adequate sample size, only individuals with decomposition codes less than 4 were included in analysis (see Leopold, 2015). Measurement error was not estimated.

#### 2.1.2 Foraging range

Foraging range was defined as the geographical range in which a stranded porpoise could have foraged. Note that this is different from the realized foraging area, which includes a component of predator “choice” regarding prey availability, which we want to avoid.

Estimating the foraging range of porpoises prior to stranding is difficult due to the unknown location of death. It is possible that a stranded porpoise was alive and swimming until just before it stranded, or carcasses could have drifted at sea for a considerable period of time (Peltier et al., 2013). This introduces uncertainty in defining the area where porpoises likely foraged. We used information on the rate at which porpoises could have moved prior to stranding to obtain informed estimates of their potential foraging range.

The foraging range was estimated using telemetry data from satellite‐linked tags deployed on harbour porpoises in the Kattegat, Belt Seas, and Western Baltic between 1997 and 2015 (see Teilmann et al. (2007) and Sveegaard et al. (2011) for tagging procedures, tag settings, and data filtering). The movements of harbour porpoises in the Kattegat and Belt Seas differ from those further north in the Skagerrak and in the North Sea (Sveegaard et al., 2011). To ensure the data were as representative as possible for porpoises that stranded in the southern North Sea, data from the southern Kattegat and further south (south of latitude 57.30°N and east of longitude 9.37°E) were excluded.

The use of stomach content data to estimate prey consumption depends on knowledge of the temporal window within which porpoises could have obtained their last meal, which is dependent on how long prey remains stay in the stomach. In the absence of information on passage rates of hard prey remains for harbour porpoises, information for similar sized gray seals *Halichoerus grypus* and harbour seals *Phoca vitulina*, which consume similar prey species, was used. Two days after consumption >50% of all otoliths were recovered in gray seal (Grellier & Hammond, 2006) and >85% in harbour seals scats (Wilson et al., 2017). To estimate harbour porpoise foraging range, a minimum timeframe of 2 days was chosen. Additionally, timeframes of 4, 6, and 8 days were applied to explore how resilient the results were to variation in the likely foraging area, including to accommodate any drifting of carcasses postmortem.

**FIGURE 1:**
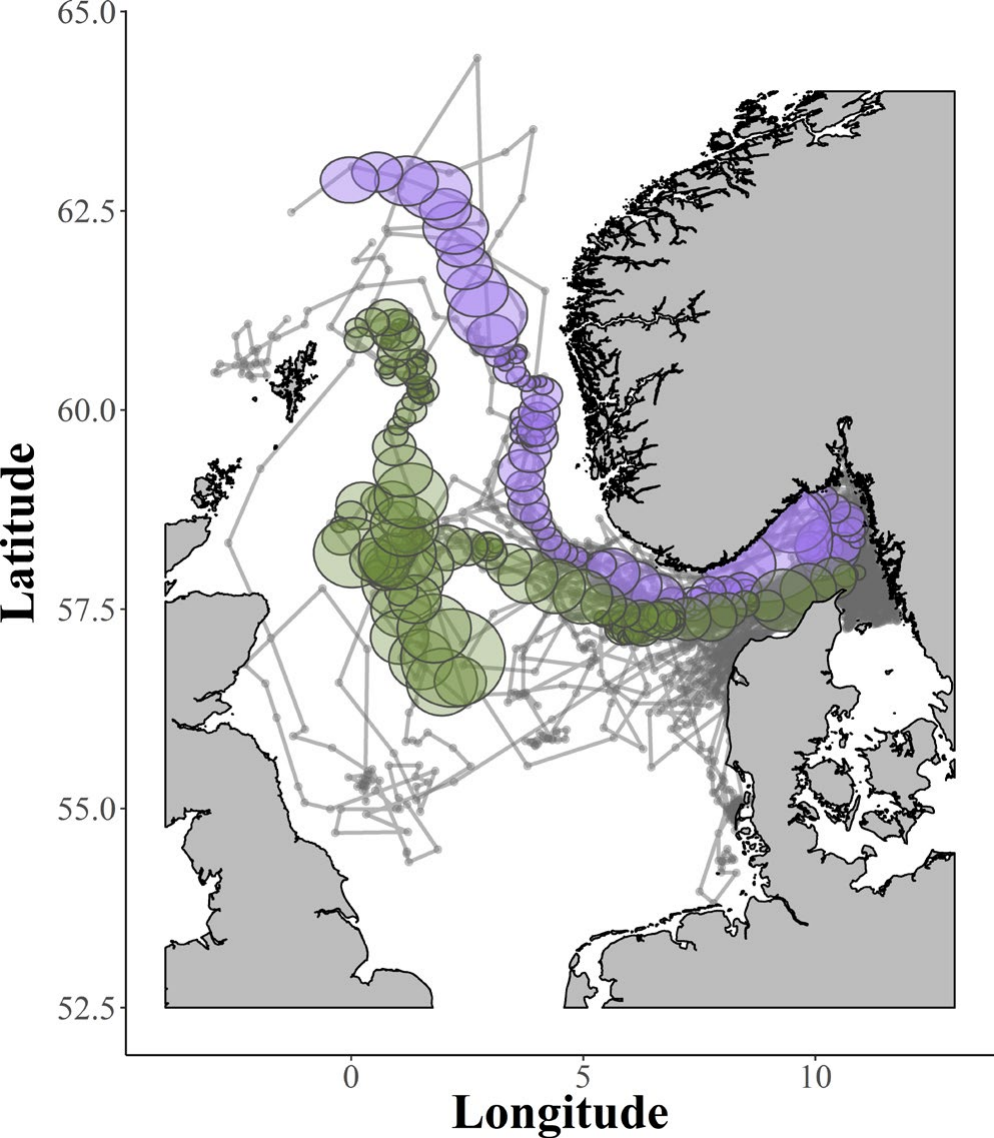
Track lines excluding Inner Danish Waters (south of latitude 57.30 and east of 9.37) of tagged harbour porpoises 1997–2015. For illustrative purposes, we show minimum enclosing circles (MECs) for two of the tagged harbour porpoises with a two‐day time frame. The green circles are the MECs for a juvenile male during the winter of 2003 and the purple circles are for a juvenile male during the summer of 2001. Grey dots represent the ARGOS positions of all porpoises

Prior to modeling the telemetry data, the track line of each tagged porpoise was processed to create positions at regular intervals. These positions were used to generate minimal enclosing circles (MECs) from sets of consecutive points for timeframes of 2, 4, 6, or 8 days (Figure 1). Using a generalized linear model (GLM), the MEC diameter (response variable assumed to follow a gamma distribution with log link) was modeled as a function of timeframe and age, sex, season (quarter of the year), and all two‐way interactions. Model selection was based on AIC scores. The variance inflation factor (VIF) was used to detect multicollinearity using a threshold of 4 (Hair et al., 2010).

Tagged individuals are measured repeatedly, so a generalized linear mixed model (GLMM) including a random effect for individual was also investigated. However, the GLM was better supported than the GLMM according to AIC scores and log‐likelihoods.

**FIGURE 2:**
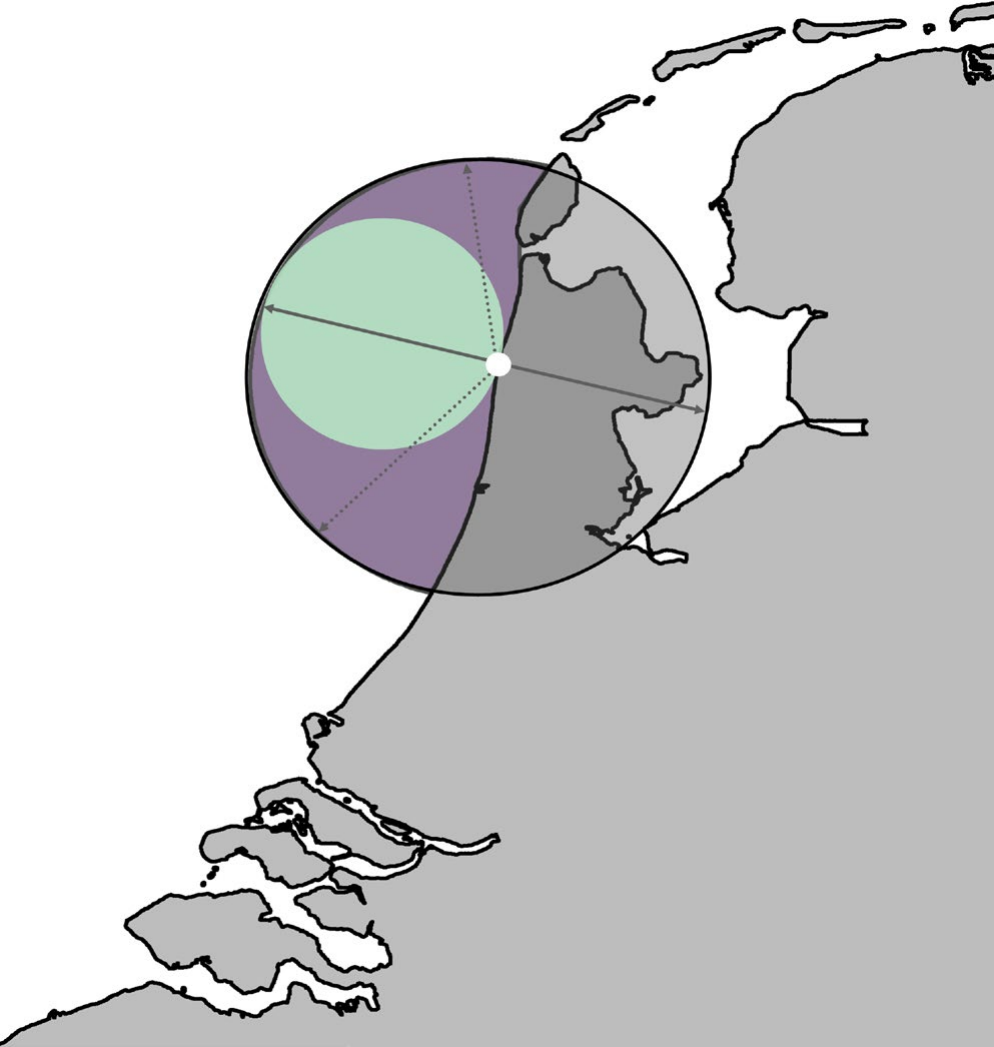
Predicted foraging range prior to stranding assuming 2 days foraging using telemetry data. Green buffer denotes the estimated minimum enclosing circle (MEC) from Danish telemetry data. White circle is the stranding location of one porpoise. Purple buffer represents the predicted foraging range prior to stranding. The diameter of the green buffer was used as the radius of the purple buffer

Stranded porpoises are located on the coast, so the diameter of the MEC estimated from the GLM was used to predict the radius of a circular buffer, centered on stranding location, to approximate the foraging range (at sea) prior to stranding for each stranded individual (Figure 2). Uncertainty about foraging range was explored by fitting separate MSFR models (see Section 2.2) for each timeframe (2, 4, 6, 8 days).

#### 2.1.3 Prey availability

Relative fish abundances were estimated using data from the North Sea International Bottom Trawl Survey (NS‐IBTS), available from the International Council for the Exploration of the Sea (ICES) (datras.ices.dk). NS‐IBTS data were available for quarter 1 (January–March) and quarter 3 (July–September).

Only size classes determined to be consumable by harbour porpoises (<40 cm—Aarefjord et al., 1995) were selected. Catch per unit effort was transformed into biomass per unit effort (BPUE in g) by applying the length–weight relationship:

(1) $$\text{BPUE}=\sum_{\text{all}\;\text{L}<\text{400 mm}}\text{a}{\left(\left(\frac{\text{L}}{\text{10}}\right)+\left(\text{0.5e}\right)\right)}^{\text{b}}\times {\text{CPUE}}_{\text{L}}$$

where *L* is length class (in mm), indicated by the lower limit of that class, *e* is the resolution of the length, either 5 or 10 mm (depending on species), CPUE*_L_* is the catch per unit effort for length class *L*, and *α* and *b* are length–weight conversion parameters, the values of which were derived from Wilhelms (2013).

**FIGURE 3:**
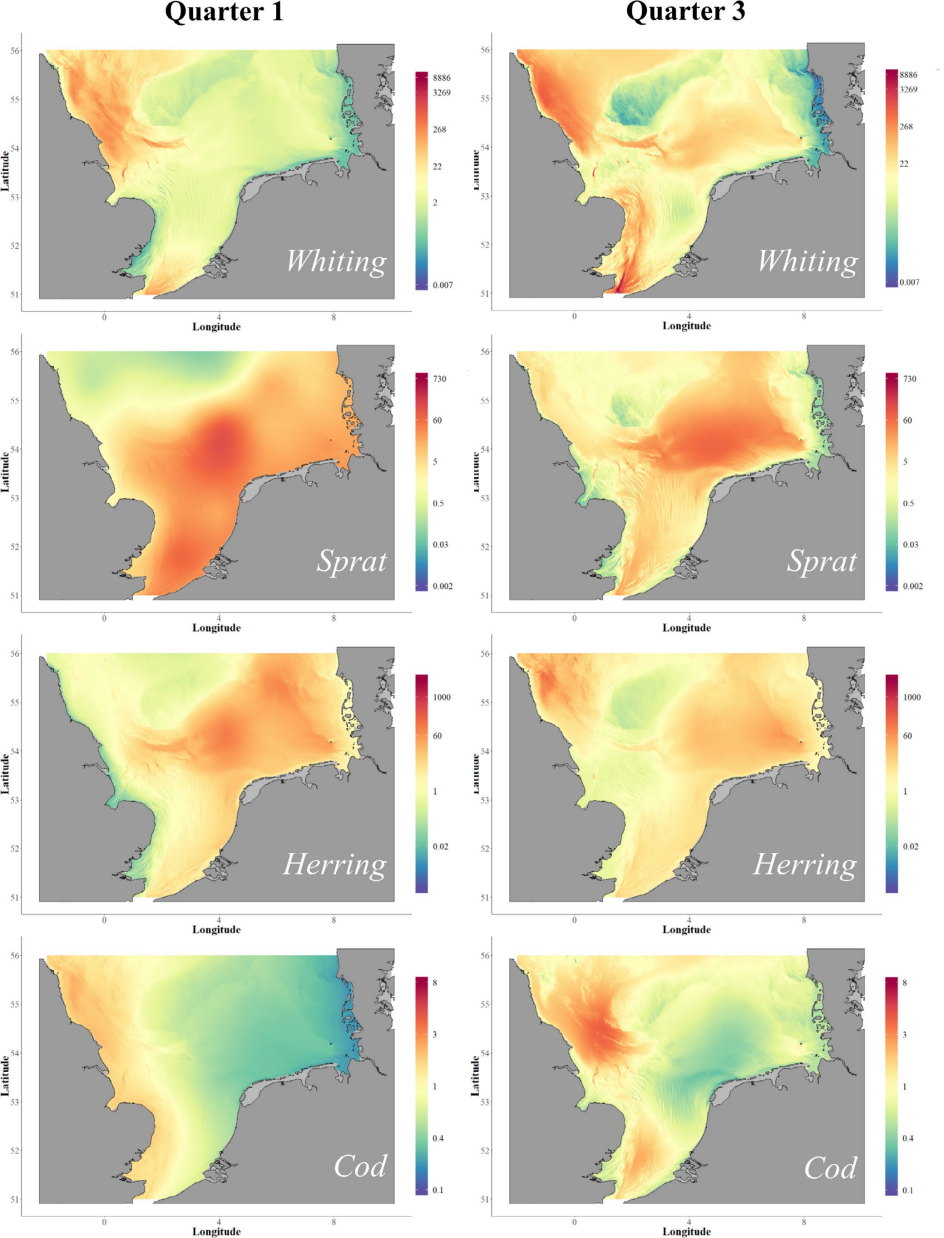
The spatial distribution of porpoise prey species for the southern North Sea illustrated for the year 2007, for Quarter one (January‐March) and Quarter three (July‐September). Density surfaces were produced from generalised additive models that were fitted to biomass per unit effort data (kg per half hour trawl, based on NS‐IBTS catches). Note different scaling along *Y*‐axes

Generalized additive models (GAMs) were used to predict distribution for each prey species over the entire southern North Sea (south of 56°N latitude (Figure 3)). The response variable BPUE, log‐transformed to reduce the effects of relatively high/low catches, was assumed to have a Gaussian error distribution. Covariates considered were longitude, latitude, depth, and year. Smoothing parameter selection was performed by restricted maximum likelihood (REML) (Wood, 2011). The model allowed the spatial pattern to change with time, by including a three‐dimensional tensor product smooth for geographical space and year:

(2) $$\text{Log}\left({\text{BPUE}}_{\mathrm{it}}\right)=s\left({\text{depth}}_{\mathrm{it}}\right)+te\left({\text{longitude}}_{i},{\text{latitude}}_{i},{\text{year}}_{t}\right)$$

For a given haul, the biomass per unit effort is represented by ${\text{BPUE}}_{\text{i}\text{,}\text{t}}$ having space coordinates *i* and a date/time *t*.

To avoid smoothing being adversely impacted by land boundaries we applied a soap film smoother (Wood et al., 2008). In generating the soap film, knots were placed over the data and land was set to zero which ensured smoothing toward data points and avoided predicting over the boundary. Comparing the soap film smoother with a conventional thin‐plate regression spline smoother showed that the soap film improved the prediction of fish densities in areas with closely adjacent land boundaries (e.g., the Strait of Dover).

The predictions of the fitted model represent expected BPUE values. To estimate the true underlying fish biomass, predictions would need to be scaled using gear efficiency and catchability estimates. However, absolute estimates of fish biomass are not required to fit a MSFR (see Section 2.2).

Sandeels are not well represented in the NS‐IBTS due to catchability issues. Therefore, for this species we used ICES estimates of sandeel spawning‐stock biomass from other data sources, including commercial catches and dredge surveys (ICES, 2016). Gobies (Gobiidae) had to be excluded because they are almost absent in the NS‐IBTS data due to their small sizes (Knijn et al., 1993), and there is no other source of data.

The relative availability, and associated uncertainty, of each main prey species to each porpoise prior to stranding was estimated as the relative amount of prey present within the area of sea within the estimated circular buffer (see Section 2.1.2). For each buffer, the *SD* of the availability of each prey species was obtained by parametric resampling the estimated coefficients from the fitted GAMs.

### 2.2 Multi‐species functional response

#### 2.2.1 Model development

A general equation allowing a single species functional response to take the form of a type I, II, or III is (Holling, 1959):

(3) $$\text{c}=\frac{{\text{αN}}^{\text{m}}}{\text{1}+{\text{αtN}}^{\text{m}}}$$

where *c* is the predator consumption rate, α is the attack rate, *N* is prey availability, *t* is the consumption/handling time, and *m* is a shape parameter.

The equation can be rewritten to include multiple prey species:

(4) $${\text{c}}_{\text{i}}=\frac{{\text{α}}_{\text{i}}{\text{N}}_{\text{i}}^{\text{m}}}{\text{1}+\sum_{\text{j}=\text{1}}^{\text{Z}}{\text{α}}_{\text{j}}{\text{t}}_{\text{j}}{\text{N}}_{\text{j}}^{\text{m}}}$$

where $c_{i}$ is the consumption of prey species *i*; ${\text{α}}_{\text{i}}$ and ${\text{t}}_{\text{i}}$ are the attack rate and handling time of species *i*. There is a total of *Z* prey species in the system. We do not have information on harbour porpoise consumption rates, but the equation can be revised in terms of diet composition:

(5) $$\frac{{\text{c}}_{\text{i}}}{\sum_{\text{j}}{\text{c}}_{\text{j}}}=\frac{{\text{α}}_{\text{i}}{\text{N}}_{\text{i}}^{\text{m}}}{\sum_{\text{j}}{\text{α}}_{\text{j}}{\text{N}}_{\text{j}}^{\text{m}}}$$

where $\sum_{\text{j}}{\text{c}}_{\text{j}}$ is the sum of the consumptions of all prey species by the predator. Not all species in the diet need to be included for this relationship to hold. Note that the denominator is identical for all prey types and then for any subset of prey $\left\{\text{1, 2 ... p}\right\}$ such that p<Z

(6) $$\frac{{\text{c}}_{\text{i}}}{\sum_{k=1}^{p}{\text{c}}_{\text{k}}}=\frac{\frac{{\text{α}}_{\text{i}}{\text{N}}_{\text{i}}^{\text{m}}}{\text{1+}\sum_{\text{j = 1}}^{\text{Z}}{\text{α}}_{\text{j}}{\text{N}}_{\text{j}}^{\text{m}}}}{\sum_{\text{k = 1}}^{\text{p}}\left(\frac{{\text{α}}_{\text{k}}{\text{N}}_{\text{k}}^{\text{m}}}{\text{1+}\sum_{\text{j = 1}}^{\text{Z}}{\text{α}}_{\text{j}}{\text{N}}_{\text{j}}^{\text{m}}}\right)}=\frac{{\text{α}}_{\text{i}}{\text{N}}_{\text{i}}^{\text{m}}}{\sum_{\text{k = 1}}^{\text{p}}{\text{α}}_{\text{k}}{\text{N}}_{\text{k}}^{\text{m}}}$$

It is therefore possible to model proportions of the diet of some prey relative to one another, leaving out some species. This is important because gobies had to be excluded even though they are important constituents of the diet (see Sections 2.1.3 and 3.1).

Catchability ${\text{q}}_{\text{i}}$ relates the survey catch of each prey species ${\text{B}}_{\text{i}}$ to the true abundance or biomass in the sea, ${\text{N}}_{\text{i}}$:

(7) $${\text{N}}_{\text{i}}={\text{q}}_{\text{i}}\times {\text{B}}_{\text{i}}$$

so Equation (5) can be rewritten as:

(8) $$\frac{{\text{c}}_{\text{i}}}{\sum_{j}{\text{c}}_{\text{j}}}=\frac{\alpha_{i}{\left(q_{i}B_{i}\right)}^{m}}{\sum_{j}{\text{α}}_{\text{j}}{\left({\text{q}}_{\text{j}}{\text{B}}_{\text{j}}\right)}^{\text{m}}}=\frac{({\text{α}}_{\text{i}}{\text{q}}_{\text{i}}^{{\text{m}}_{\text{i}}}){\text{B}}_{\text{i}}^{\text{m}}}{\sum_{j}(\alpha_{j}q_{j}^{m_{j}})B_{j}^{m}}$$

Then, defining constant ${\text{a}}_{\text{i}}={\text{α}}_{\text{i}}{\text{q}}_{\text{i}}^{{\text{m}}_{\text{i}}}$ we can write:

(9) $$\frac{{\text{c}}_{\text{i}}}{\sum_{\text{j}}{\text{c}}_{\text{j}}}=\frac{{\text{a}}_{\text{i}}{\text{B}}_{\text{i}}^{\text{m}}}{\sum_{\text{j}}{\text{a}}_{\text{j}}{\text{B}}_{\text{j}}^{\text{m}}}$$

Therefore, from diet composition and BPUE data we can estimate the value of the ${\text{a}}_{\text{i}}$ parameters without needing to correct for catchability.

#### 2.2.2 Model fitting

For model fitting, relative prey abundance was rescaled so that the maximum observed value was 100 to assist numerical performance and convergence. The estimated values of ${\text{a}}_{\text{i}}$ are thus a measure of prey “preference” or attack rate in relation to an index of abundance and not to absolute estimates of biomass.

The shape parameter *m* determines how sigmoidal the response is and thus influences the form of the functional response. For a hyperbolic type II functional response, the shape parameter *m* = 1. If *m* > 1, the functional response defines a sigmoidal type III functional response (Real, 1977). We compared two model types: a hyperbolic type II functional response with shape parameter *m* = 1 (model 1) and a sigmoidal type III functional response with *m* = 1.5 (model 2).

The relationship between relative prey availability and consumption was estimated for each main prey species in turn by setting the availability of all other prey to one of three specific constant levels (minimum, mean, and maximum).

Markov chain Monte Carlo (MCMC) methods used for model fitting enabled uncertainty in diet composition and prey availability estimates to be incorporated. At each step in the Markov chain, for each prey species, random values of relative prey availability were drawn from a zero‐truncated normal distribution. For each model, the MCMC was run for 10,000 iterations after a burn‐in of 1000 samples with two parallel Markov chains.

Prey species that contributed <5% to the diet of harbour porpoises were grouped into a single category “other prey.” All goby species were added to this category because no prey availability estimates for these species could be calculated (see Section 2.1.3).

It is difficult to create informative priors for ${\text{a}}_{\text{i}}$ because diet composition data allow relative but not absolute values of attack rate ${\text{a}}_{\text{i}}$ to be estimated (Equation 9 holds if all the ${\text{a}}_{\text{i}}$ are multiplied by any arbitrary constant). Consequently, to estimate relative values for *α*, a wide uniform prior U(0,10) was used for all prey species except sandeels, for which attack rate was fixed at a value of 1. This allowed for the relative values of attack rate *a* of other prey species to be larger or smaller than for sandeels. The marginal posterior distributions of *α* were checked after model fitting, to confirm that they had very low weights toward the prior's upper limit of 10, to ensure that the uniform priors were not over‐constraining the exploration of parameter space. After fitting, models were compared using DIC scores (Spiegelhalter et al., 2002).

### 2.3 Model prediction

To illustrate the model's ability to predict consumption under different regimes of prey availability, the estimated parameters of the best fitting model were used to predict diet composition in 2011 (a year of high sandeel spawning‐stock biomass, SSB, in the southern North Sea) and 2020 (a year of low sandeel SSB, and the most current advice from ICES for all prey species considered), assuming similar prey distribution and porpoise stranding locations. Prey availability of 2011 was rescaled using estimates of SSB from the ICES Stock Assessment Database for 2020, following Smout et al. (2014). Changes in diet composition were estimated relative to the daily biomass or energy consumption of an average adult male porpoise (i.e., 1.7 kg or 6.7 MJ per day (Gallagher et al., 2018)). Estimates from the literature were used to convert biomass to energetic content (Table 5). Energy values for gobies were used for the “other” prey category because they were the most prevalent species in that group.

## 3 RESULTS

### 3.1 Diet composition

Stomach content data were available from 455 harbour porpoises. Juveniles of both sexes (*n* = 344 (74.8%)) dominated the sample. The “main” prey species included six different types of fish: whiting (27.1% by biomass), gobies (20.8%), and sandeels (18.5%) were the most dominant species. Lesser contributions were made by herring (8.5%), sprat (*Sprattus sprattus*) (6.9%), and cod (*Gadus morhua*) (6.0%). Other species comprised 12.2% of the diet.

### 3.2 Foraging range

In total, 2448 locations of 54 harbour porpoises were included in the telemetry analysis (females: 15 juveniles, 3 adults; males: 24 juveniles, 12 adults). The number of tracking days per individual ranged from 8 to 350 days (mean = 93.9; *SD* = 87.3).

In models to estimate the diameter of minimum enclosing circles (MECs), all covariates had a VIF score lower than 1.4; therefore, multicollinearity could be disregarded. Model results are summarized in Table 1. Age, quarter, sex, and timeframe were all found to be significant predictors (*p* < .01) for the foraging range (MEC diameter) and explained 24.5% of the variation. Predicted foraging range was smaller for males than for females and for juveniles in comparison with adults. Foraging range was significantly smaller in spring in comparison with the other seasons.

**TABLE 1:** Summary of the generalized linear modeling to predict the foraging range of tagged harbour porpoises

Time frame	Months	Adult		Juvenile	
		Female	Male	Female	Male
2 days	January–March	34.9 (1.3)	32.6 (1.2)	30.5 (1.3)	28.2 (1.1)
2 days	April–June	42.3 (1.8)	39.9 (1.7)	37.9 (1.6)	35.5 (1.4)
2 days	July–September	33.2 (1.3)	30.8 (1.1)	28.8 (1.0)	26.4 (0.4)
2 days	October–December	33.5 (1.3)	31.1 (1.1)	29.1 (1.1)	26.7 (0.9)
4 days	January–March	58.4 (1.2)	56.0 (1.2)	54.0 (1.2)	51.6 (1.1)
4 days	April–June	65.8 (1.7)	63.4 (1.6)	61.4 (1.6)	59.0 (1.4)
4 days	July–September	56.6 (1.3)	54.2 (1.1)	52.2 (1.0)	49.9 (0.6)
4 days	October–December	57.0 (1.2)	54.6 (1.1)	52.6 (1.1)	50.2 (0.9)
6 days	January–March	81.9 (1.4)	79.5 (1.3)	77.5 (1.4)	75.1 (1.3)
6 days	April–June	89.2 (1.8)	86.9 (1.7)	84.8 (1.7)	82.5 (1.6)
6 days	July–September	80.1 (1.5)	77.7 (1.3)	75.7 (1.3)	73.3 (1.0)
6 days	October–December	80.4 (1.3)	78.1 (1.3)	76.0 (1.3)	73.7 (1.1)
8 days	January–March	105.3 (1.6)	102.9 (1.6)	100.9 (1.7)	98.6 (1.6)
8 days	April–June	112.7 (2.0)	110.3 (2.0)	108.3 (1.9)	105.9 (1.8)
8 days	July–September	103.5 (1.8)	101.2 (1.7)	99.1 (1.6)	96.8 (1.4)
8 days	October–December	103.9 (1.6)	101.5 (1.6)	99.5 (1.6)	97.1 (1.5)

Note

Predicted mean diameter and *SD* (in parentheses) of minimum enclosing circle (MEC) in (km) for harbour porpoises according to time frame, quarter, age, and sex.

### 3.3 Prey availability

Correlograms of the final models of relative prey abundance indicated very weak autocorrelation, and deviance residuals were evenly spread. BPUE predictions in all grid cells, including unsurveyed cells, are shown in Figure 3. The final models explained between approximately one third to two thirds of the total observed variation in the BPUE values (Table 2).

**TABLE 2:** Percentage deviance explained values for the selected generalized additive models (GAMs) of relative prey availability (BPUE) per prey species and quarter

Species	Quarter 1 (January–March)	Quarter 3 (July–September)
	% deviance explained	% deviance explained
Cod	28.3	25.3
Herring	43.1	30.3
Sprat	32.9	32.8
Whiting	60.7	54.0

**FIGURE 4:**
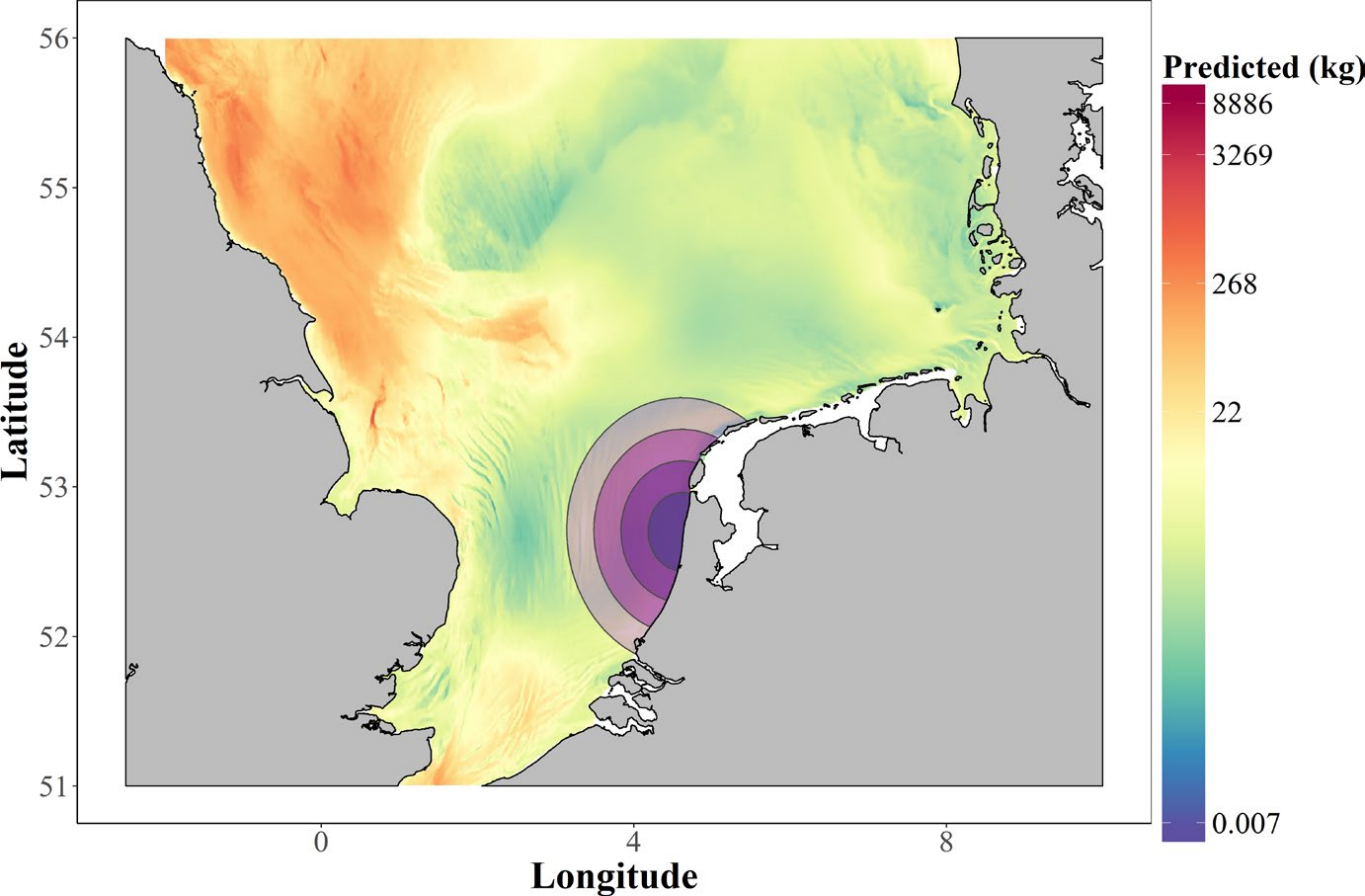
Predicted availability of whiting for a stranded harbour porpoise along the Dutch coastline in Quarter one (January‐March), 2014. Purple buffers represent different assumptions about the foraging range prior to stranding: the size of the buffers corresponds to 2, 4, 6, or 8 days spent foraging, based on our analysis of telemetry data. The coloured background denotes the predicted relative abundance of whiting in terms of biomass per unit effort values (kg per half hour trawl, based on NS‐IBTS catches)

As described above, the availability of each prey species was predicted for each individual porpoise, within the circular buffer that represented the foraging range for each timeframe (see Section 2.1.3). For illustration, Figure 4 displays the prediction of whiting availability for one porpoise for different timeframes.

### 3.4 Multi‐species functional response

The best MSFR model in terms of timeframe according to DIC scores (Table 3) was the 4 days model. Model 2 (DIC = 97,202) with a type III functional response was selected over model 1 (DIC = 117,216) with a type II functional response. Consequently, predictions are only presented for the 4 days type III functional response model.

**TABLE 3:** Deviance information criterion (DIC) scores of multi‐species functional response (MSFR) models according to different foraging range as determined by buffer sizes estimated for different time periods

Time period	2 days	4 days	6 days	8 days
DIC score type III	113,090	97,202	133,846	107,295
DIC score type II	125,161	117,216	147,177	138,544

The posterior distributions for a were well defined given the wide uniform priors (Appendix S2). The relative attack rate was considerably higher for sprat (mean = 0.238, 95% CI [0.226, 0.254]) in comparison with whiting (mean = 0.120, 95% CI [0.114, 0.129]), herring (mean = 0.101, 95% CI [0.095, 0.108]), “other prey” (mean = 0.089, 95% CI [0.085, 0.095]), and cod (mean = 0.058, 95% CI [0.055, 0.063]). Recall that these estimates are in relation to a relative attack rate for sandeel fixed at a value of 1.

Model predictions of diet composition captured the overall pattern in the observed diet (Table 4). The model predicted higher proportions of sandeels and cod and lower proportions of other species in comparison with the observed diet, but all predictions fell well within the range of uncertainty indicating that the model predictions were robust.

**TABLE 4:** Predicted diet (for the 4 days MSFR model) of harbour porpoises and observed diet derived from bootstrapping stomach content of stranded animals

Prey species	Predicted	observed
Cod	5.0 (0.5)	2.1 (10.0)
Herring	6.4 (4.0)	4.8 (10.0)
Sandeel	25.1 (8.2)	17.2 (14.2)
Sprat	7.0 (3.8)	7.7 (14.2)
Whiting	8.6 (1.8)	12.7 (15.8)
Others	47.6 (18.9)	55.0 (12.2)

Note

Expressed as mean (*SD*) percentages of total prey mass.

**FIGURE 5:**
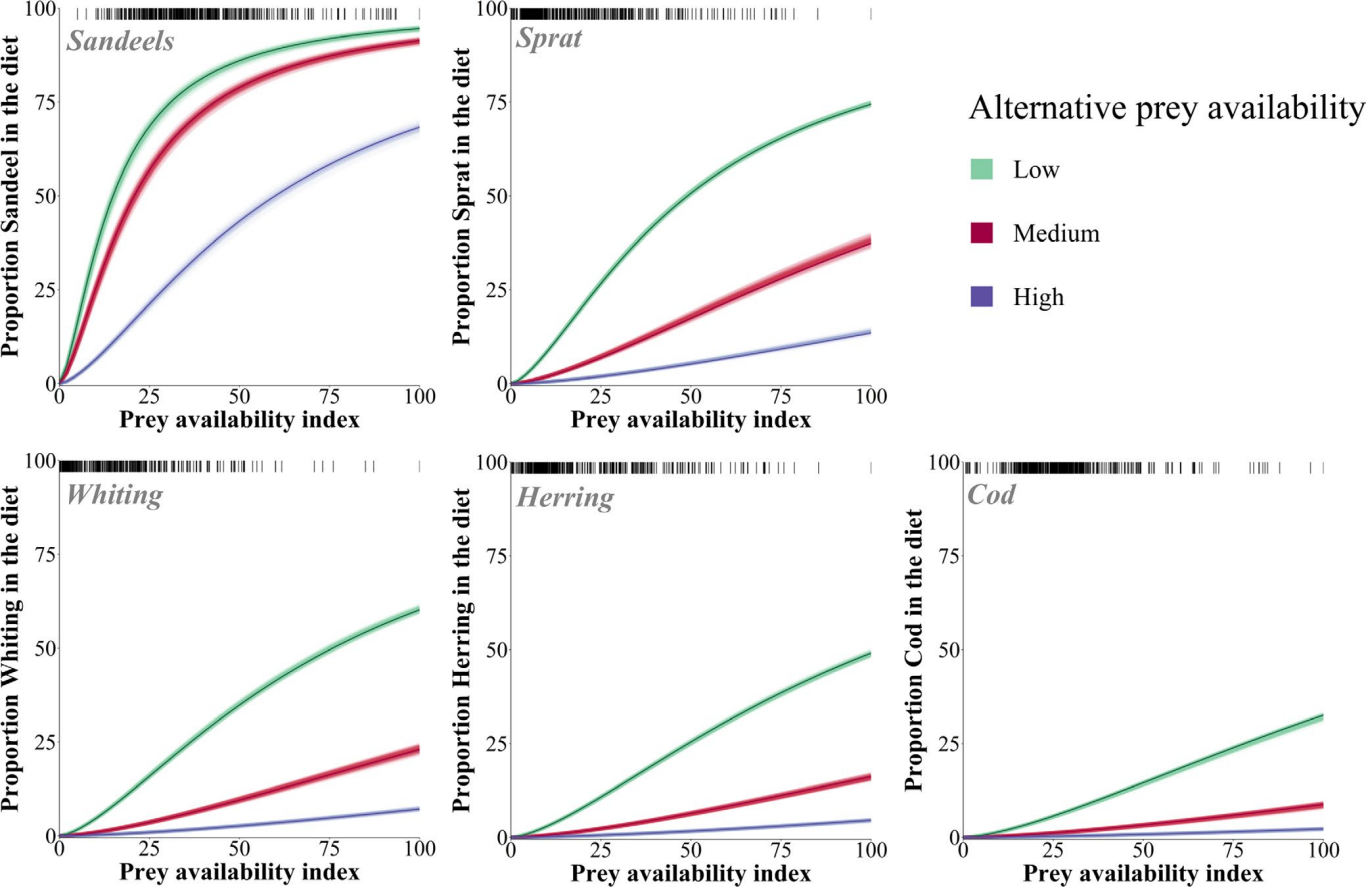
Relationship between prey availability and consumption by harbour porpoises for the 4 days MSFR model. Relationships are shown as a single‐species plot at three different levels of alternative prey (all other prey) availability

The model predicted a strong relationship between relative prey availability and relative prey consumption by harbour porpoises (Figure 5). Overall, consumption of the selected prey species decreased as more alternative prey (all other species) was available. However, the relative consumption of sandeels remained relatively high over all three levels of alternative prey availability (Figure 5).

The relative change in predicted diet composition between 2011 and 2020 was most noticeable for sandeels (−63%), whiting (+61%), herring (−56%), and sprat (+50%). The change in prey availability resulted in a relatively small predicted change of ~2% in daily consumption (either an increase in kg consumed or a reduction in energy intake). If porpoises forage to meet energy requirements, intake of biomass would have needed to increase by ~27 g per day in 2020 compared with 2011. Conversely, if porpoises forage to consume constant biomass, this would have led to a reduction in energy intake of ~163 kJ per day in 2020 in comparison with 2011. This decrease in energy for a fixed intake of biomass was because of a reduced consumption of sandeels, substituted mainly by an increase in consumption of sprat and whiting, from 2011 to 2020.

## 4 DISCUSSION

Integrating disparate datasets to model the MSFR for harbour porpoises in the southern North Sea provides a methodological framework that may be appropriate for other predators. Results from our case study show that sandeels are an important and possibly a preferred prey for harbour porpoise, thus increasing knowledge of the foraging ecology of this important marine predator.

### 4.1 Method evaluation and sensitivity

Setting suitable spatial scales can be a major challenge in ecological studies and the accuracy of any modeled relationship between prey consumption and availability is strongly dependent on achieving realistic spatio‐temporal overlap. In this study, the foraging distributions of porpoises prior to stranding (our source of diet information) are unknown, so it is crucial to explore whether assumptions made about the foraging range of these animals are reasonable. Our novel approach was to find the most likely foraging area prior to stranding by predicting the range used as a function of time period based on telemetry data, and using the MSFR model fit to determine the appropriate time period of 4 days. There is little relative difference in modeled prey distribution for each prey species in the areas where porpoises could have been foraging in the vicinity of the Dutch coast (Figures 3 and 4), indicating that the overall pattern of results is unlikely to vary much over the range of time periods modeled (2–8 days). This is confirmed by lack of variation in the emerging patterns of estimated attack rates or the shape of the functional response (Appendix S1). In this case study, our methodology thus appears rather robust to this aspect of uncertainty.

### 4.2 Ecological inference

Different shapes of the predator functional response have different implications for prey populations, especially at low prey densities. In our best fitting model with a sigmoidal type III functional response, predation mortality decreases when a prey species becomes rare and is indicative of prey switching when prey is at low abundance (that is, there is a change in preference dependent on prey abundance). This may result in persistence and/or stabilizing effects on predator–prey dynamics (Murdoch & Oaten, 1975) because it may prevent one prey species from outcompeting others (Roughgarden & Feldman, 1975). A type III response may result from a number of ecological mechanisms, including prey refuge (McNair, 1986), and learning time (Tinbergen, 1960).

Classically, the attack rate parameter a in the functional response equation can be interpreted as a form of relative preference of the predator for a certain prey type. Here, we interpret these values cautiously because of the nature of the prey abundance estimates we used. These were indices, scaled in proportion to maximum values, and they were not estimates of overall total biomass (which is difficult to calculate). Thus, for example the “maximum” value of sandeel abundance was 100 and so was the maximum value for whiting.

In this study, porpoises consumed a disproportionately larger proportion of the most abundant prey. Sandeel consumption remained high even when other prey were abundant and was considerably higher than the consumption of other prey at equal availability index values. At prey abundances similar to those available to our study animals, harbour porpoise diets often have a high proportion of sandeels (Jansen, 2013; Santos et al., 2004), and it also implies that sandeel availability might have a particularly strong effect on the consumption by porpoises of other prey species in this area. Habitat models of harbour porpoise in the North Sea have found that harbour porpoise density increases with decreasing distance to sandeel grounds (Gilles et al., 2016), suggesting that porpoises could be attracted to those areas.

Harbour porpoises in better body condition have been found to be more likely to have higher amounts of fatty fish, such as sandeels, in their diet (Leopold, 2015). Our results add to the body of evidence that sandeels are important to porpoises. Sandeels have high energy content and are abundant in the southern North Sea, forming an important forage fish resource that supplies a number of predator species including harbour porpoises, seabirds (Rindorf et al., 2000), and gray and harbour seals (Wilson & Hammond, 2019).

Despite considerable differences in predicted diet composition in 2011 and 2020 (Table 5), differences in predicted consumption were relatively small (~2%), illustrating little variation in overall biomass or energy intake. However, if energy‐rich prey species (i.e., clupeids and sandeels) were reduced to low levels, this could result in porpoises needing to increase biomass consumed to avoid failing to meet their energetic requirements.

**TABLE 5:** Predicted change in harbour porpoise diet composition in terms of percentages of total prey biomass (%M) and energy (%E) for 2011 and 2020, and prey energy density

Species	%M 2011	%M 2020	%E 2011	%E 2020	Prey energy (kJ g^−1^)
Cod	5.9	3.8	5.0	3.3	4.2^a^
Herring	4.1	1.8	5.1	2.3	6.2^b^
Sandeel	26.6	9.8	31.2	11.7	5.8^c^
Sprat	3.8	9.5	5.8	14.9	7.6^c^
Whiting	9.5	15.3	8.3	13.6	4.3^b^
Others	50.1	59.8	44.6	54.2	4.4^d^

^a^ Lawson et al. (1997).

^b^ Pedersen and Hislop (2001).

^c^ Wanless et al. (2005).

^d^ Plimmer (1921).

Sandeel abundance in the northwestern North Sea has declined since 2000 (MacDonald et al., 2019). Poor seabird breeding success in the northwest North Sea has been linked to a reduction in the availability and quality of sandeels (Wanless et al., 2005, 2018). Our results confirming the importance of sandeels to harbour porpoise and indicating their possible preference for this prey are consistent with the reduction of sandeel biomass in the northern North Sea being a driver of the distributional shift of porpoises from the northern to the southern North Sea between 1994 and 2005 (Hammond et al., 2013). However, this needs further exploration of the impact of other potential drivers such as competition with other sandeel predators (i.e., sea birds, other marine mammals, foraging fish as well as fisheries).

### 4.3 Data limitations

Foraging range was estimated from telemetry data collected in areas of the North Sea outside the study area. By including data only from the area believed to be most similar to the study area, we sought to minimize error in estimated foraging range. Estimates of foraging range using movement data are uncertain and conservative. Active swimming is faster than drifting, so true foraging range will be larger than that estimated from drifting alone. The fitted MSFRs gave similar results for different assumptions about the foraging area available to porpoises before stranding, so we conclude that the lack of telemetry data from the study area should not affect our conclusions appreciably. ARGOS data from telemetry tags are subject to location error which was not quantified in this study but is believed to be negligible in this context.

Our prey availability estimates assume that relative prey abundance is proportional to true abundance. We also assume that prey abundance reflects prey availability to predators; however, the relationship between prey abundance and prey availability is largely unknown, not least because differences in prey behavior (e.g., diurnal and seasonal variation in schooling and burying behavior) may affect this. Our methodology is appropriate if spatio‐temporal trends in relative abundance reflect those in absolute abundance of prey, which seems a reasonable assumption.

Most fisheries surveys, including the North Sea IBTS, sample at a coarse spatial and temporal resolution. Some species, especially sandeels and gobies in this study, are poorly sampled. Given the importance of sandeels for many marine predators (Engelhard et al., 2013; Gilles et al., 2016; Wanless et al., 2005; Wilson & Hammond, 2019) and the lack of knowledge regarding spatio‐temporal variability in their distribution and abundance, improving effective sampling and modeling of sandeel distribution would improve the quality of the inferences made from future studies. The inability to model sandeel and goby distributions spatially could have led to error in availability estimates, especially because sandeel distribution is extremely patchy (Wright et al., 2000) and largely unknown for gobies. The importance of gobies could have been underestimated because they were excluded from the prey availability analysis. Although information on goby distribution and abundance is largely lacking, gobies are extremely abundant within Dutch coastal waters (Tulp et al., 2008). Therefore, it might be reasonable to assume that these species have a relatively consistent availability.

Care should be taken in making inferences from stranding data because they do not represent an unbiased sample of the population; there is likely an over‐representation of individuals that are inexperienced, old, and/or in poor health (Pierce et al., 2004). Indeed, a large proportion of the stranded individuals in this study were juveniles so our results are biased toward this age class. Thus, we do not know to what extent our results reflect the functional response of a “typical” porpoise. Most information on cetacean diet derives from stomach contents analysis. Using diet data from stomachs of animals that have been by‐caught in fishing nets, or even killed by gray seals or bottlenose dolphins (van Neer et al., 2020; Ross & Wilson, 1996), would be an alternative way to look at harbour porpoise diet. However, the diet of by‐caught porpoises diet could be biased toward target species of the fishery and “net selection” of inexperienced individuals (Santos & Pierce, 2003), and porpoises killed by seals or dolphins may be more vulnerable to predation and not representative of the population.

The predicted diet for 2011 and 2020 for an adult male porpoise assumed sampling the same porpoise stranding locations, fish distributions having the same pattern scaled by North Sea wide stock assessment estimates, and that the relationship between fish biomass and energy was constant for each species. However, these assumptions could be violated in several ways. For example, the distribution of porpoises and/or fish could have changed, there are differences in porpoise prey consumption according to sex, age (Booth, 2020; Leopold, 2015), and prey energy densities vary by size class, season, etc. (Pedersen & Hislop, 2001). Therefore, a more elaborate analysis is required to explore the impact of changing these factors on the predictions.

### 4.4 Context and applications

Applying a Bayesian approach to model the MSFR appears to work well, allowing incorporation of uncertainty in prey availability and consumption estimates. These features, together with the resilience of the results, suggest that the modeled MSFR provides a strong methodological framework that can be applied (generalized) to a range of other species and might aid in quantifying the ecological role of other predators that consume a variety of prey. For example, similar data exist for seabirds (Wanless et al., 2005), gray seals, and harbour seals in the North Sea (Carter et al., 2020; Wilson & Hammond, 2019) and applying this framework could provide valuable new insights into their population dynamics, especially in the context of possible competition for prey between these two seal species (Wilson & Hammond, 2019). To take this further, the MSFR could be integrated into ecosystem models to predict and test how prey and predator populations are expected to change under different fisheries management and climatic scenarios that impact prey availability. This could also shed light on the extent of direct and indirect competition between marine mammals, seabirds, and fisheries and possibly on the outcomes of fisheries management and stock recovery programs.

## CONFLICT OF INTEREST

The authors declare no conflict of interest.

## AUTHOR CONTRIBUTIONS

**Janneke M. Ransijn:** Conceptualization (equal); formal analysis (lead); methodology (equal); visualization (lead); writing‐original draft (lead); writing‐review & editing (equal). **Philip S. Hammond:** Conceptualization (equal); methodology (equal); supervision (equal); writing‐review & editing (equal). **Mardik F. Leopold:** Data curation (equal); writing‐review & editing (equal). **Signe Sveegaard:** Data curation (equal); writing‐review & editing (equal). **Sophie C. Smout:** Conceptualization (equal); methodology (equal); supervision (equal); writing‐review & editing (equal).

## Supporting information

Appendix S1‐S2

## Data Availability

Data to estimate the relative availability of prey species are openly available from ICES webpage (datras.ices.dk). Data and code for modelling the MSFR are deposited in the Dryad Digital Repository: https://doi.org/10.5061/dryad.z8w9ghxdg.

## References

[ece38380-bib-0001] Aarefjord, H. , Bjorge, A. J. , Kinze, C. C. , & Lindstedt, I. (1995). Diet of harbour porpoise (*Phocoena* *phocoena*) in Scandinavian waters. Report of the International Whaling Commission, 16, 211–222.

[ece38380-bib-0002] Booth, C. G. (2020). Food for thought: Harbor porpoise foraging behavior and diet inform vulnerability to disturbance. Marine Mammal Science, 36, 195–208. 10.1111/mms.12632

[ece38380-bib-0003] Carter, M. I. D. , Boehme, L. , Duck, C. D. , Grecian, J. , Hastie, G. D. , McConnell, B. J. , Miller, D. L. , Morris, C. , Moss, S. , Thompson, P. , & Russell, D. J. F. (2020). Habitat‐based predictions of at‐sea distribution for grey and harbour seals in the British Isles (pp. 74). Sea Mammal Research Unit, University of St Andrews, Report to BEIS, OESEA‐16‐76/OESEA‐17‐78.

[ece38380-bib-0004] Dale, B. W. , Adams, L. G. , & Bowyer, R. T. (1994). Functional response of wolves preying on barren‐ground caribou in a multiple‐prey ecosystem. Journal of Animal Ecology, 63, 644–652. 10.2307/5230

[ece38380-bib-0005] Engelhard, G. H. , Blanchard, J. L. , Pinnegar, J. K. , van der Kooij, J. , Bell, E. D. , Mackinson, S. , & Righton, D. A. (2013). Body condition of predatory fishes linked to the availability of sandeels. Marine Biology, 160, 299–308. 10.1007/s00227-012-2088-1

[ece38380-bib-0006] Estes, J. A. , Terborgh, J. , Brashares, J. S. , Power, M. E. , Berger, J. , Bond, W. J. , Carpenter, S. R. , Essington, T. E. , Holt, R. D. , Jackson, J. B. C. , Marquis, R. J. , Oksanen, L. , Oksanen, T. , Paine, R. T. , Pikitch, E. K. , Ripple, W. J. , Sandin, S. A. , Scheffer, M. , Schoener, T. W. , … Wardle, D. A. (2011). Trophic downgrading of planet Earth. Science, 333, 301–306. 10.1126/science.1205106 21764740

[ece38380-bib-0007] Gallagher, C. A. , Stern, J. S. , & Hines, E. (2018). The metabolic cost of swimming and reproduction in harbor porpoises (*Phocoena* *phocoena*) as predicted by a bioenergetic model. Marine Mammal Science, 34, 875–900. 10.1111/mms.12487

[ece38380-bib-0008] Gilles, A. , Viquerat, S. , Becker, E. A. , Forney, K. A. , Geelhoed, S. C. V. , Haelters, J. , Nabe‐Nielsen, J. , Scheidat, M. , Siebert, U. , Sveegaard, S. , van Beest, F. M. , van Bemmelen, R. , & Aarts, G. (2016). Seasonal habitat‐based density models for a marine top predator, the harbor porpoise, in a dynamic environment. Ecosphere, 7, e01367. 10.1002/ecs2.1367

[ece38380-bib-0009] Grellier, K. , & Hammond, P. S. (2006). Robust digestion and passage rate estimates for hard parts of grey seal (*Halichoerus* *grypus*) prey. Canadian Journal of Fisheries and Aquatic Sciences, 63, 1982–1998. 10.1139/f06-092

[ece38380-bib-0010] Hair, J. , Black, W. C. , Babin, B. J. , & Anderson, R. E. (2010). Multivariate data analysis (7th ed.). Pearson Education International.

[ece38380-bib-0011] Hammond, P. S. , Lacey, C. , Gilles, A. , Viquerat, S. , Borjesson, P. , Herr, H. , Macleod, K. , Ridoux, V. , Santos, M. B. , Scheidat, M. , Telimann, J. , Vingada, J. , & Øien, N. (2021). Estimates of cetacean abundance in European Atlantic waters in summer 2016 from the SCANS‐III aerial and shipboard surveys (pp. 42). Sea Mammal Research Unit, University of St Andrews.

[ece38380-bib-0012] Hammond, P. S. , Berggren, P. , Benke, H. , Borchers, D. L. , Collet, A. , Heide‐Jørgensen, M. P. , Heimlich, S. , Hiby, A. R. , Leopold, M. F. , & Øien, N. (2002). Abundance of harbour porpoise and other cetaceans in the North Sea and adjacent waters. Journal of Applied Ecology, 3, 361–376. 10.1046/j.1365-2664.2002.00713.x

[ece38380-bib-0013] Hammond, P. S. , Macleod, K. , Berggren, P. , Borchers, D. L. , Burt, L. , Cañadas, A. , Desportes, G. , Donovan, G. P. , Gilles, A. , Gillespie, D. , Gordon, J. , Hiby, L. , Kuklik, I. , Leaper, R. , Lehnert, K. , Leopold, M. , Lovell, P. , Øien, N. , Paxton, C. G. M. , … Vázquez, J. A. (2013). Cetacean abundance and distribution in European Atlantic shelf waters to inform conservation and management. Biological Conservation, 164, 107–122. 10.1016/j.biocon.2013.04.010

[ece38380-bib-0014] Hammond, P. S. , & Rothery, P. (1996). Application of computer sampling in the estimation of seal diet. Journal of Applied Statistics, 23, 525–533. 10.1080/02664769624062

[ece38380-bib-0015] Holling, C. S. (1959). The components of predation as revealed by a study of small mammal predation of the European pine sawfly. Canadian Entomologist, 91, 293–320. 10.4039/Ent91293-5

[ece38380-bib-0016] ICES (2016). Report of the benchmark on Sandeel (WKSand 2016), 31 October–4 November 2016, Bergen, Norway (301 pp). ICES CM 2016/ACOM:33.

[ece38380-bib-0017] ICES (2018). Report of the International Bottom Trawl Survey Working Group (IBTSWG), 19 ‐ 23 March 2018, Oranmore, Ireland (233 pp). ICES CM 2018/EOSG:01.

[ece38380-bib-0018] Jansen, O. E. (2013). Fishing for food, feeding ecology of harbour porpoises *Phocoena* *phocoena* and white‐beaked dolphins *Lagenorhynchus* *albirostris* in Dutch waters. PhD thesis. Wageningen University, The Netherlands.

[ece38380-bib-0019] Kastelein, R. A. , Helder‐Hoek, L. , Jennings, N. , van Kester, R. , & Huisman, R. (2019). Reduction in body mass and blubber thickness of harbor porpoises (*Phocoena* *phocoena*) due to near‐fasting for 24 hours in four seasons. Aquatic Mammals, 45, 37–47. 10.1578/AM.45.1.2019.37

[ece38380-bib-0020] Knijn, R. J. , Boon, T. , Heessen, H. , & Hislop, J. (1993). Atlas of North Sea fishes: Based on bottom‐trawl survey data for the years 1985‐1987. ICES Cooperative Research Report, 194, 268, ICES, Copenhagen.

[ece38380-bib-0021] Lawson, J. W. , Hare, J. A. , Noseworthy, E. , & Friel, J. K. (1997). Assimilation efficiency of captive ringed seals (*Phoca* *hispida*) fed different diets. Polar Biology, 18, 107–111. 10.1007/s003000050164

[ece38380-bib-0022] Leopold, M. F. (2015). Eat and be eaten, Porpoise diet studies. PhD thesis. Wageningen University, The Netherlands.

[ece38380-bib-0023] Lunn, D. , Thomas, A. , Best, N. , & Spiegelhalter, D. (2000). WinBUGS ‐ A Bayesian modelling framework: Concepts, structure, and extensibility. Statistics and Computing, 10, 325–337. 10.1023/A:1008929526011

[ece38380-bib-0024] MacDonald, A. , Speirs, D. C. , Greenstreet, S. P. R. , Boulcott, P. , & Heath, M. R. (2019). Trends in sandeel growth and abundance off the east coast of Scotland. Frontiers in Marine Science, 6, 201. 10.3389/fmars.2019.00201

[ece38380-bib-0025] McNair, J. N. (1986). The effects of refuges on predator‐prey interactions: A reconsideration. Theoretical Population Biology, 29, 38–63. 10.1016/0040-5809(86)90004-3 3961711

[ece38380-bib-0026] Morozov, A. , & Petrovskii, S. (2013). Feeding on multiple sources: Towards a universal parameterization of the functional response of a generalist predator allowing for switching. PLoS One, 8, e74586. 10.1371/journal.pone.0074586 24086356 PMC3783441

[ece38380-bib-0027] Murdoch, W. W. , & Oaten, A. (1975). Predation and population stability. Advances in Ecological Research, 9, 1–131. 10.1016/S0065-2504(08)60288-3

[ece38380-bib-0028] Pedersen, J. , & Hislop, J. R. G. (2001). Seasonal variations in the energy density of fishes in the North Sea. Journal of Fish Biology, 59, 380–389. 10.1111/j.1095-8649.2001.tb00137.x

[ece38380-bib-0029] Peltier, H. , Baagøe, H. J. , Camphuysen, K. C. J. , Czeck, R. , Dabin, W. , Daniel, P. , Deaville, R. , Haelters, J. , Jauniaux, T. , Jensen, L. F. , Jepson, P. D. , Keijl, G. O. , Siebert, U. , Van Canneyt, O. , & Ridoux, V. (2013). The stranding anomaly as population indicator: The case of harbour porpoise *Phocoena* *phocoena* in North‐Western Europe. PLoS One, 8, e62180. 10.1371/journal.pone.0062180 23614031 PMC3632559

[ece38380-bib-0030] Pierce, G. J. , Santos, M. B. , Learmonth, J. A. , Mente, E. , & Stowasser, G. (2004). Methods for dietary studies on marine mammals. Investigating the roles of cetaceans in marine ecosystems. CIESM Workshop Monographs, 25, 29‐36, Monaco.

[ece38380-bib-0031] Plimmer, R. H. A. (1921). Analyses and energy values of foods.

[ece38380-bib-0032] R Development Core Team (2018). R: A language and environment for statistical computing. R Foundation for Statistical Computing. http://www.R‐project.org

[ece38380-bib-0033] Real, L. A. (1977). The kinetics of functional response. ‐American Naturalist, 111, 289–300. 10.1086/283161

[ece38380-bib-0034] Rindorf, A. , Wanless, S. , & Harris, M. P. (2000). Effects of changes in sandeel availability on the reproductive output of seabirds. Marine Ecology Progress Series, 202, 241–252. 10.3354/meps202241

[ece38380-bib-0035] Rojano‐Doñate, L. , McDonald, B. I. , Wisniewska, D. M. , Johnson, M. , Teilmann, J. , Wahlberg, M. , Højer‐Kristensen, J. , & Madsen, P. T. (2018). High field metabolic rates of wild harbour porpoises. Journal of Experimental Biology, 221, jeb185827. 10.1242/jeb.185827 30523043

[ece38380-bib-0036] Ross, H. M. , & Wilson, B. (1996). Violent interactions between bottlenose dolphins and harbour porpoises. Proceedings of the Royal Society of London. Series B: Biological Sciences, 263, 283–286. 10.1098/rspb.1996.0043

[ece38380-bib-0037] Roughgarden, J. , & Feldman, M. (1975). Species packing and predation pressure. Ecology, 56, 489–492. 10.2307/1934982

[ece38380-bib-0038] Santos, M. B. , & Pierce, G. J. (2003). The diet of harbour porpoise (*Phocoena* *phocoena*) in the northeast Atlantic. Oceanography and Marine Biology: an Annual Review, 41, 355–390.

[ece38380-bib-0057] Santos, M. B. , Pierce, G. J. , Learmonth, J. A. , Reid, R. J. , Ross, H. M. , Patterson, I. A. P. , Reid, D. G. , & Beare, D. (2004). Variability in the diet of harbor porpoises (*Phocoena phocoena*) in Scottish waters 1992‐2003. Marine Mammal Science, 20, 1–27.

[ece38380-bib-0039] Smout, S. , Rindorf, A. , Hammond, P. S. , Harwood, J. , & Matthiopoulos, J. (2014). Modelling prey consumption and switching by UK grey seals. ICES Journal of Marine Science, 71, 81–89. 10.1093/icesjms/fst109

[ece38380-bib-0040] Spiegelhalter, D. J. , Best, N. G. , Carlin, B. P. , & van der Linde, A. (2002). Bayesian measures of model complexity and fit. Journal of the Royal Statistical Society: Series B (Statistical Methodology), 64, 583–639. 10.1111/1467-9868.00353

[ece38380-bib-0041] Spitz, J. , Trites, A. W. , Becquet, V. , Brind'Amour, A. , Cherel, Y. , Galois, R. , & Ridoux, V. (2012). Cost of living dictates what whales, dolphins and porpoises eat: The importance of prey quality on predator foraging strategies. PLoS One, 7, e50096. 10.1371/journal.pone.0050096 23185542 PMC3503768

[ece38380-bib-0042] Suryawanshi, K. R. , Redpath, S. M. , Bhatnagar, Y. V. , Ramakrishnan, U. , Chaturvedi, V. , Smout, S. C. , & Mishra, C. (2017). Impact of wild prey availability on livestock predation by snow leopards. Royal Society Open Science, 4, 170026. 10.1098/rsos.170026 28680665 PMC5493907

[ece38380-bib-0043] Sveegaard, S. , Teilmann, J. , Tougaard, J. , Dietz, R. , Mouritsen, K. N. , Desportes, G. , & Siebert, U. (2011). High‐density areas for harbor porpoises (*Phocoena* *phocoena*) identified by satellite tracking. Marine Mammal Science, 27, 230–246. 10.1111/j.1748-7692.2010.00379.x

[ece38380-bib-0044] Teilmann, J. , Larsen, F. , & Desportes, G. (2007). Time allocation and diving behaviour of harbour porpoises (*Phocoena* *phocoena*) in Danish and adjacent waters. Journal of Cetacean Research and Management, 9, 201–210.

[ece38380-bib-0045] Tinbergen, L. (1960). The natural control of insects in pine‐woods. 1. Factors influencing the intensity of predation by songbirds. Archives Néerlandica Zoologica, 13, 266–336.

[ece38380-bib-0046] Tulp, I. , Bolle, L. J. , & Rijnsdorp, A. D. (2008). Signals from the shallows: In search of common patterns in long‐term trends in Dutch estuarine and coastal fish. Journal of Sea Research, 60, 54–73. 10.1016/j.seares.2008.04.004

[ece38380-bib-0047] van Neer, A. , Gross, S. , Kesselring, T. , Grilo, M. L. , Ludes‐Wehrmeister, E. , Roncon, G. , & Siebert, U. (2020). Assessing harbour porpoise carcasses potentially subjected to grey seal predation. Scientific Reports, 10, 1–9. 10.1038/s41598-020-80737-9 33004890 PMC7530704

[ece38380-bib-0048] Wanless, S. , Harris, M. P. , Newell, M. A. , Speakman, J. R. , & Daunt, F. (2018). Community‐wide decline in the occurrence of lesser sandeels *Ammodytes* *marinus* in seabird chick diets at a North Sea colony. Marine Ecology Progress Series, 600, 193–206. 10.3354/meps12679

[ece38380-bib-0049] Wanless, S. , Harris, M. P. , Redman, P. , & Speakman, J. R. (2005). Low energy values of fish as a probable cause of a major seabird breeding failure in the North Sea. Marine Ecology Progress Series, 294, 1–8. 10.3354/meps294001

[ece38380-bib-0050] Wilhelms, I. (2013). Atlas of length‐weight relationships of 93 fish and crustacean species from the North Sea and the North‐East Atlantic. Thünen Working Paper, 12, 1:552. Thünen Institute of Sea Fisheries, Hamburg.

[ece38380-bib-0051] Wilson, L. J. , Grellier, K. , & Hammond, P. S. (2017). Improved estimates of digestion correction factors and passage rates for harbor seal (*Phoca* *vitulina*) prey in the northeast Atlantic. Marine Mammal Science, 33, 1149–1169. 10.1111/mms.12436

[ece38380-bib-0052] Wilson, L. J. , & Hammond, P. S. (2019). The diet of harbour and grey seals around Britain: Examining the role of prey as a potential cause of harbour seal declines. Aquatic Conservation: Marine and Freshwater Ecosystems, 29, 71–85. 10.1002/aqc.3131

[ece38380-bib-0053] Wisniewska, D. M. , Johnson, M. , Teilmann, J. , Rojano‐Doñate, L. , Shearer, J. , Sveegaard, S. , Miller, L. A. , Siebert, U. , & Madsen, P. T. (2016). Ultra‐high foraging rates of harbor porpoises make them vulnerable to anthropogenic disturbance. Current Biology, 26, 1441–1446. 10.1016/j.cub.2016.03.069 27238281

[ece38380-bib-0054] Wood, S. N. (2011). Fast stable restricted maximum likelihood and marginal likelihood estimation of semiparametric generalized linear models. Journal of the Royal Statistical Society: Series B (Statistical Methodology), 73, 3–36. 10.1111/j.1467-9868.2010.00749.x

[ece38380-bib-0055] Wood, S. N. , Bravington, M. V. , & Hedley, S. L. (2008). Soap film smoothing. Journal of the Royal Statistical Society: Series B (Statistical Methodology), 70, 931–955. 10.1111/j.1467-9868.2008.00665.x

[ece38380-bib-0056] Wright, P. J. , Jensen, H. , & Tuck, I. (2000). The influence of sediment type on the distribution of the lesser sandeel, *Ammodytes* *marinus* . Journal of Sea Research, 44, 243–256. 10.1016/S1385-1101(00)00050-2

