# Supplementary material for: Integrating disparate datasets to model the functional response of a marine predator: A case study of harbour porpoises in the southern North Sea
Source: Ecol Evol. 2021 Nov 30;11(23):17458–70. doi: 10.1002/ece3.8380 (PMC8668753; doi:10.1002/ece3.8380)
Supplement: Supplementary file 1 — Appendix S1‐S2 [file ECE3-11-17458-s001.docx]

# Supporting Information

**Appendix S1.**

**Table S1.** Attack rate according to different foraging ranges. The posterior mean and 95% Bayesian credible intervals of attack rate *a*, for all “main” prey species and different foraging ranges (represented by different timeframes). Recall that the attack rate for sandeel was set at a fixed value of 1 in the model.

| **Prey species** | **Foraging range (timeframe)** | | | |
| --- | --- | --- | --- | --- |
|  | 2 days | 4 days | 6 days | 8 days |
| Cod | 0.046  [0.040, 0.053] | 0.058  [0.055, 0.063] | 0.055  [0.052, 0.058] | 0.055  [0.053, 0.058] |
| Herring | 0.101  [0.095, 0.107] | 0.101  [0.095, 0.108] | 0.104  [0.099, 0.110] | 0.100  [0.093, 0.106] |
| Sprat | 0.227 [0.214,0.241] | 0.238  [0.226, 0.254] | 0.207  [0.197, 0.220] | 0.224  [0.219, 0.238] |
| Whiting | 0.116  [0.109, 0.122] | 0.120  [0.114, 0.129] | 0.106  [0.101, 0.113] | 0.115  [0.109, 0.121] |
| Others | 0.082  [0.079, 0.087] | 0.089  [0.085, 0.095] | 0.095  [0.091, 0.103] | 0.090  [0.085, 0.094] |

**Fig. S1.**  Functional response shapes to different foraging ranges. Relationship between prey availability and consumption by harbour porpoises to different foraging ranges. Relationships are shown as a single-species plot at three different levels of alternative prey (all other prey) availability. (A) assumed 2 days of foraging prior to stranding, (B) 6 days and (C) 8 days.


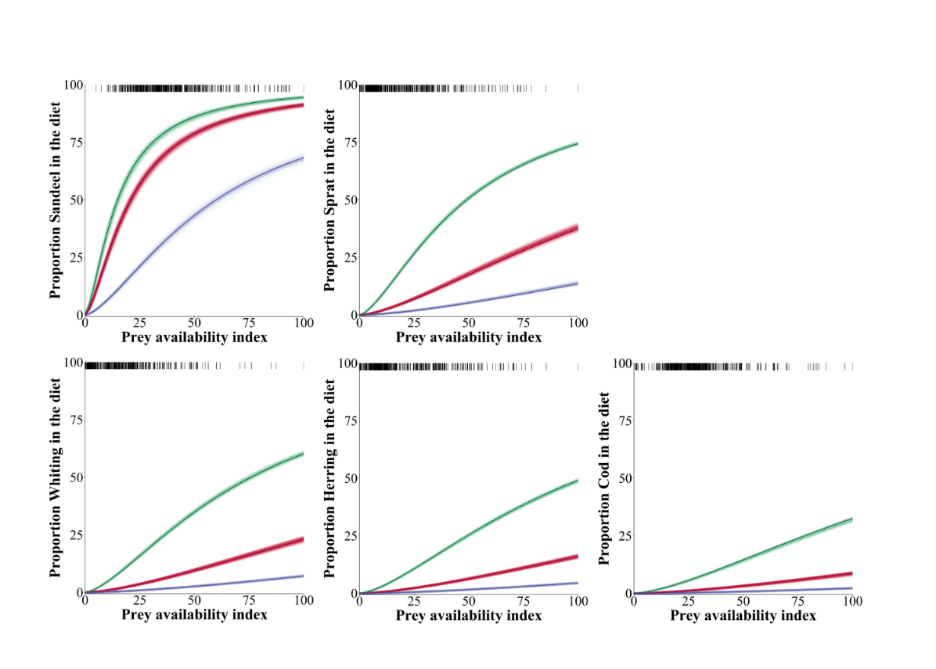


**A**


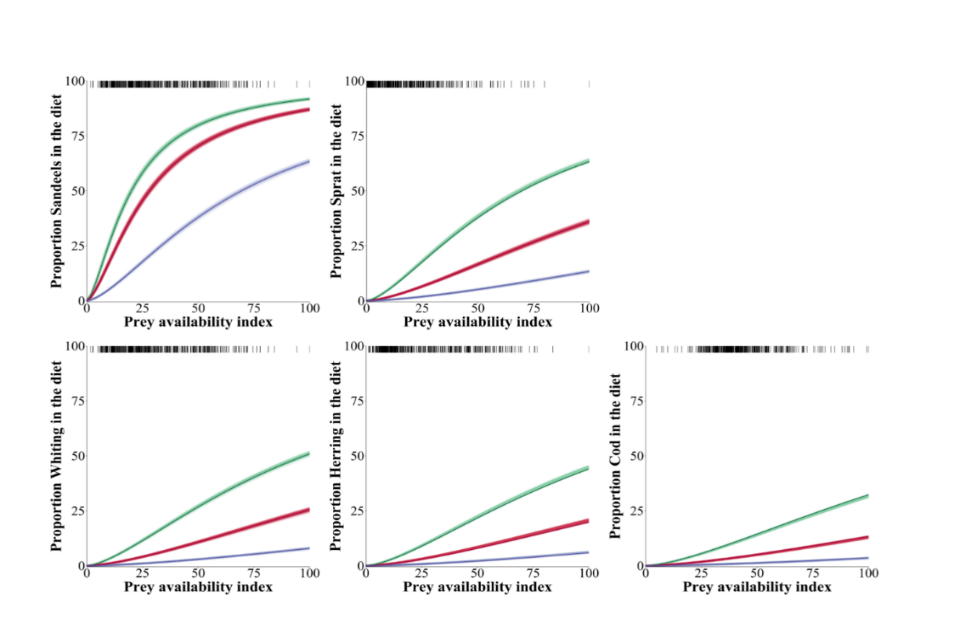


**C**


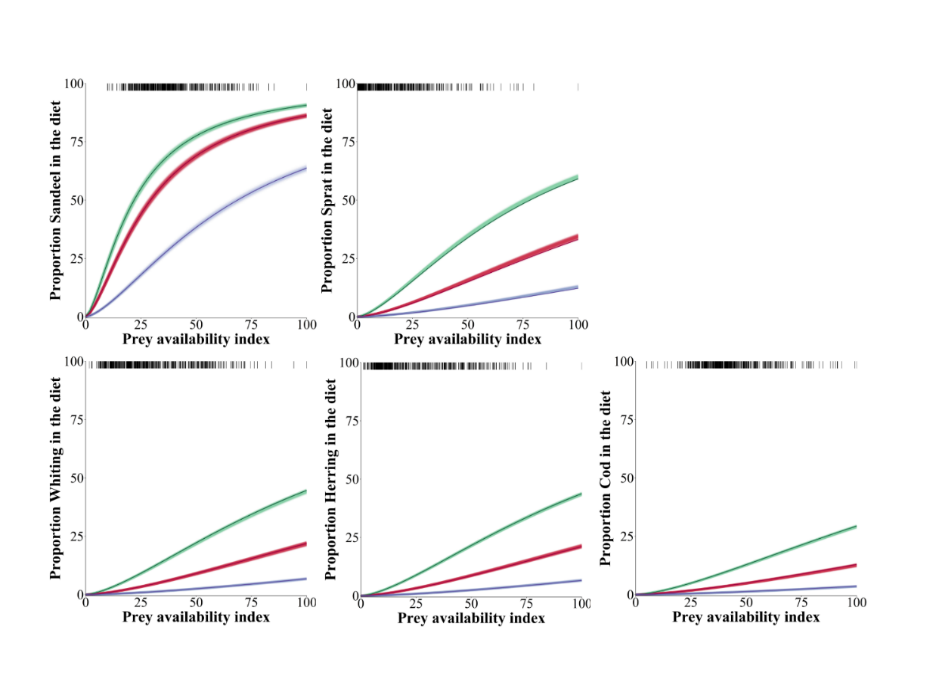


**B**


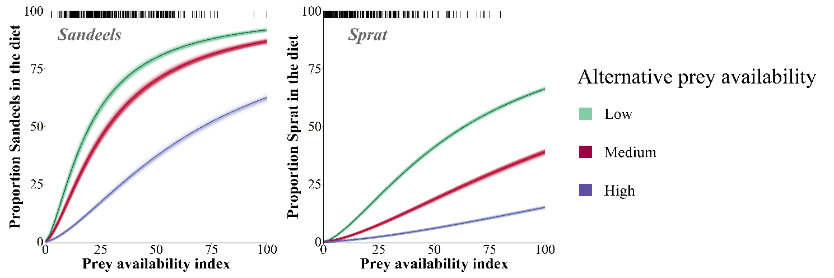

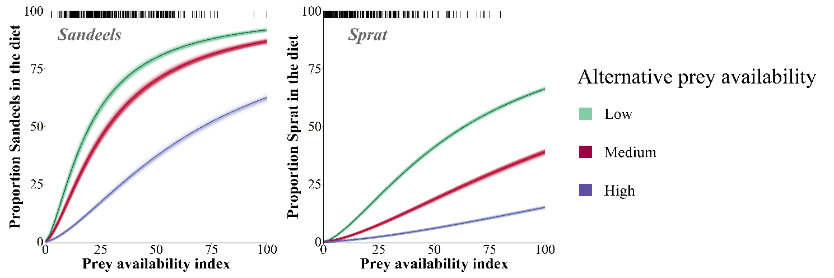

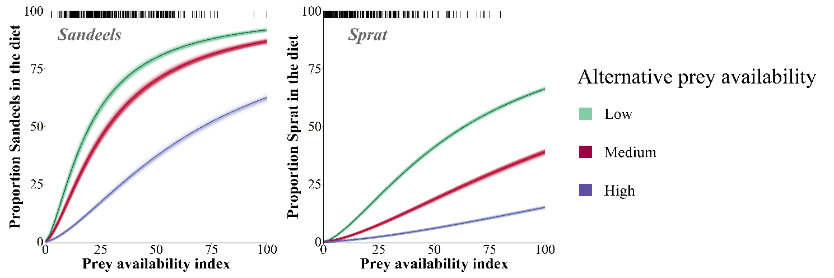


**Appendix S2.**


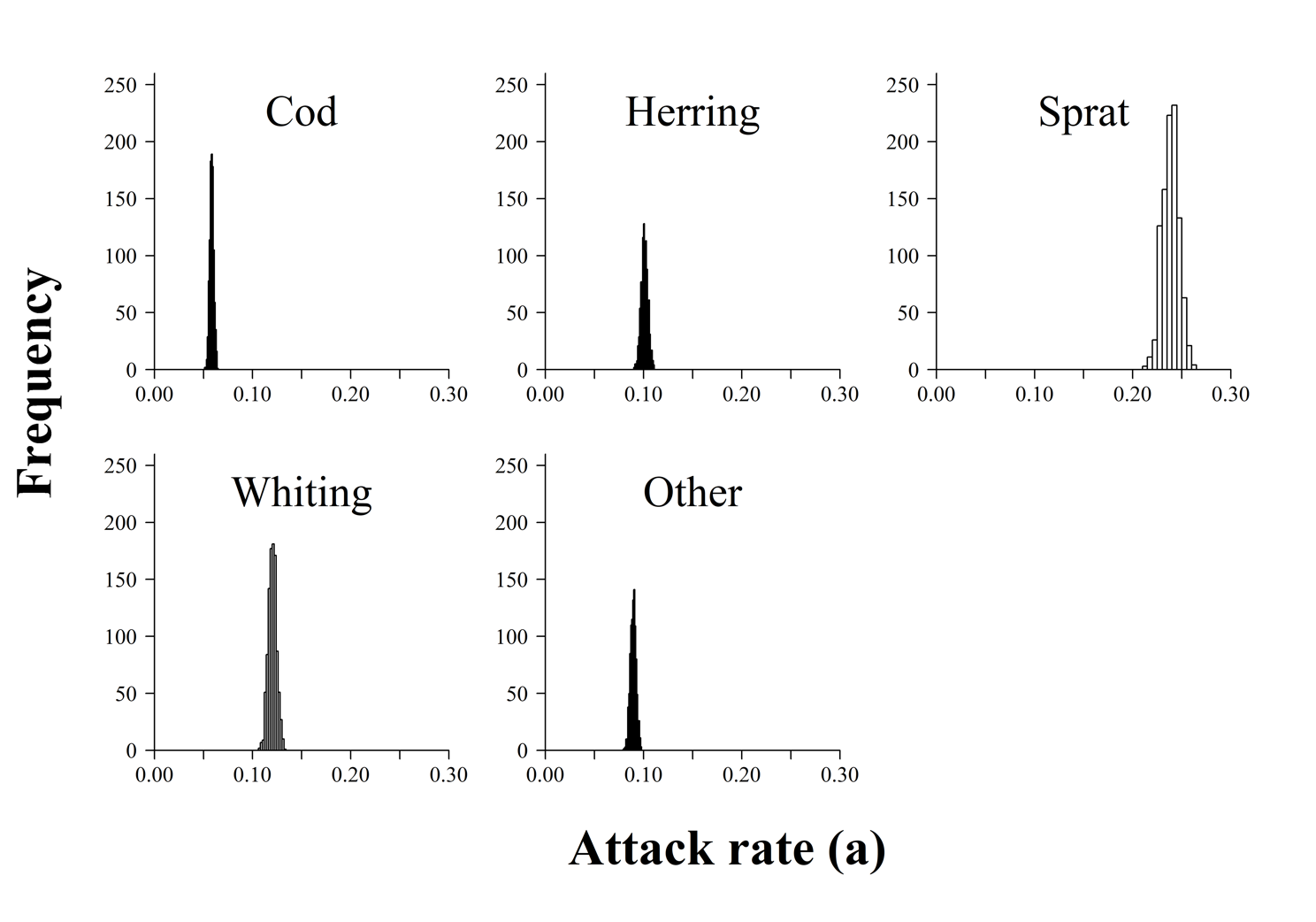
**Fig. S2.**  Posterior distribution for the attack rates for the porpoise prey species in our model (*a*). Sandeels are not displayed as they were set at a fixed value of 1.
